# Exploring the Psychological Effects and Physical Exertion of Using Different Movement Interactions in Casual Exergames That Promote Active Microbreaks: Quasi-Experimental Study

**DOI:** 10.2196/55905

**Published:** 2024-08-26

**Authors:** Aseel Berglund, Helena Orädd

**Affiliations:** 1 Department of Computer and Information Science Linköping University Linköping Sweden

**Keywords:** physical activity, exergames, casual exergames, enjoyment, exertion, motion-based games

## Abstract

**Background:**

Prolonged sedentary behavior, such as sitting or reclining, has consistently been identified as a stand-alone risk factor for heightened cardiometabolic risk and overall mortality. Conversely, interrupting sedentary periods by incorporating short, active microbreaks has been shown to mitigate the negative effects of sedentary behavior. Casual exergames, which mix elements of casual gaming with physical activity, are one prospective intervention to reduce sedentary behavior because they require physical exertion. Casual exergames have shown promise in fostering emotional and physical advantages when played in specific circumstances. However, little research exists on how different types of movement interactions impact the psychological effects as well as the physical exertion of playing casual exergames.

**Objective:**

The primary aim of this work was to explore the psychological effects and physical exertion of playing casual exergames lasting 2 minutes. More precisely, the investigation focused on comparing upper body and full body movement interactions. In addition, the work examined variations in body positions, considering both standing and seated positions during upper body movement interactions.

**Methods:**

Two casual exergames were developed and investigated through 2 quasi-experimental studies. In study 1, we investigated how players’ perceptions of control, exertion, and immersion were affected by using upper body as opposed to full body exergame controllers when playing casual exergames. In study 2, we investigated differences in positive affect, performance, enjoyment, and exertion when playing casual exergames with upper body movement interactions in seated and standing positions.

**Results:**

Study 1 showed that perceived control was significantly higher for upper body movement interactions than for full body movement interactions (*P*=.04), but there were no significant differences regarding perceived exertion (*P*=.15) or immersion (*P*=.66). Study 2 showed that positive affect increased significantly for both standing (*P*=.003) and seated (*P*=.001) gameplay. The participants in the standing gameplay group showed slightly higher actual exertion; however, there were no differences between the groups in terms of positive affect, perceived exertion, enjoyment, or performance.

**Conclusions:**

Casual exergames controlled by upper body movement interactions in seated gameplay can produce similar psychological effects and physical exertion as upper body movement interactions in standing gameplay and full body movement interactions. Therefore, upper body and seated casual exergames should not be overlooked as a suitable microbreak activity.

## Introduction

### Background

High levels of sedentary behavior (such as sitting or lying down) [1] and low levels of moderate to vigorous physical exercise are characteristics of an inactive lifestyle [1,2]. The people most vulnerable to the negative effects of an inactive lifestyle are those who have highly sedentary lives with minimal exercise [3,4]. The advances in domestic and workplace technologies, along with the changes in personal and public transportation, have decreased the necessity for physical activity, leading to increased sedentary lifestyles among populations [5]. The frequency of light-intensity activities (such as walking and performing household chores) in outdoor and nonoffice occupations has decreased in the last few decades [5]. Sedentary behavior is a widespread issue affecting various groups of people (eg, children [6-8], youth [9], and adults [10,11]) across different demographics and settings. While watching television, reading, using a computer, and playing video games are discretionary activities [12], sitting during school [6-8], university [9], or work [10,11] hours is generally nondiscretionary [12]. Therefore, researchers have investigated the impact of sedentary behavior [5]. Numerous studies have demonstrated the connection between excessive sedentary behavior and unfavorable health indicators or results [13-15], regardless of how much time is spent exercising to counteract the sedentary behavior [16]; for instance, when physical activity is taken into account, adults who sit 10 hours a day, as opposed to 1 hour a day, still show a 34% higher risk of increased mortality [17].

Incorporating active microbreaks has demonstrated the potential to mitigate the adverse consequences linked to sedentary behavior [14,18] and improve people’s moods [19]; for example, 2- to 3-minute microbreaks with a light activity level every 30 minutes can positively impact mental and physical health [20-22]. Another study showed that over the course of 8 weeks, individuals who were encouraged to stand up and move every 30 minutes for 1 to 2 minutes during the workday significantly decreased their sedentary behavior by approximately 36 minutes per workday [23]. Thus, encouraging frequent, short breaks from sitting could improve health outcomes related to the risk of chronic diseases [23].

Exergames—video games that use movement and physical exertion during gameplay—have been shown to be effective in providing a good psychological experience [24] while also offering advantages for the player’s health [25]. Exergames have been created with a variety of health-related goals in mind, including rehabilitation [26] and better mental health [27]. Exergames can increase physical activity [28,29], decrease sedentary behavior [30-33], and promote more active breaks [34-37]. Exergaming has been shown to achieve both moderate [31,38] and vigorous [38] levels of exertion, suggesting that exergames have the potential to serve as an alternative to conventional exercise [39].

Casual exergames are easily learned and quickly accessible exergames with simple rules, designed to motivate players to engage in moderate-intensity exercise during short play sessions [33]. Incorporating active microbreaks using casual exergames has demonstrated the potential to interrupt sedentary behavior [32,37,40]. Playing casual exergames can also induce positive affective states [31,32,41], which can reduce stress levels [42] and enhance overall well-being [43]. Previous studies have demonstrated that casual exergames can be enjoyable and generate appropriate levels of exertion [31,32,40]. However, little research has been conducted on the psychological experience and physical exertion when playing casual exergames [44-73] or on using different types of movement interactions in casual exergames [45].

When developing exergames, it is important to consider both the psychological and physiological aspects of the player’s experience [46-48]. According to the dual flow model, the attractiveness of an exergame is determined by balancing the player’s skill level with the exergame’s challenge level, resulting in a state of psychological flow; and the effectiveness of the exergame is determined by balancing the player’s fitness level with the exergame’s physical intensity level [35,49]. As the optimal psychological experience and the optimal physiological experience are not necessarily aligned, focusing exclusively on boosting in-game experience may risk reducing health-related advantages [48]. On the basis of the dual flow model, movements with great psychological attractiveness and physiological effectiveness should be used [48]. The psychological and physiological aspects are also connected because the level of enjoyment experienced during playing exergames is correlated with increased exertion levels [44].

One aspect of psychological attractiveness in the exergame experience is immersion, defined in the context of video games as the degree to which the player participates in a game [50] and loses awareness of their surroundings while playing [51]. Immersion is closely related to the concept of flow, which is defined as the psychological state in which one is fully involved in an activity, losing self-consciousness [52]. Immersion impacts the psychological attractiveness of exergames [53] and can be achieved when players have control over the game’s actions [50]. While both flow and immersion revolve around player engagement in a game, flow pertains to the general motivation and enjoyment of an activity, while immersion is more directly tied to the user experience and can be seen as a quantifiable aspect of the game experience that relates to both flow and motivation [44]. Movement-based controllers can enhance immersion levels by allowing for natural interaction, leading to increased well-being associated with physical exercise [53]. As control is one of the first stages in creating an immersive experience, it should be considered as a factor that might affect the player’s immersive experience and, in turn, the casual exergame’s psychological attractiveness [50]. Higher control can also contribute to improved performance, which is positively related to game enjoyment [54-56].

The characteristics of players’ movements when exergaming are influenced by the characteristics of the exergame, the difficulty of remaining motivated without sacrificing the quality of the movements, the exergaming experience even when it is of short duration, and the scoring of points [57]. For the physical exertion of playing exergames, the type of movement interaction used in the exergame is an important design decision [47,58]. One decision revolves around using upper body or full body movements. Specifically, for casual exergames designed to target upper body movements, it is worth investigating whether the use of upper body movements can encourage sufficient levels of exertion. To induce positive health-related outcomes, it has previously been stated that casual exergame play should produce a moderate level of exertion [33]. Previous research has shown that upper body movements in exergames have lower potential for increasing exertion compared to full body movements [46,59]. A meta-analysis discovered that lower body and full body exergames produced more energy expenditure than upper body exergames; thus, the authors concluded that upper body exergaming movements are insufficient for achieving adequate energy expenditure [23]. Another study measured movements during gameplay of 3 different exergames using upper body and full body movements and discovered that adequate physical activity required full body movements [58]. However, another meta-analysis showed that exergames with continuous upper body movements have the potential to meet the recommendations for a moderate-intensity activity level [39]; for example, playing Wii boxing exergames with upper body movement interactions has been shown to elicit a moderate-intensity activity level [39,60-63]. On the basis of the mixed results of previous studies, it is worth further investigating whether upper body movements can elicit similar levels of exertion as full body movements for casual exergames as well as how the psychological experience is affected.

Another factor that can affect the psychological and physiological aspects of casual exergames is whether the exergame is designed to be played in a seated or standing position. One study comparing seated and standing gameplay in an exergame for individuals with mobility impairments found that the participants playing seated had higher perceived exertion [64]. However, because the participants chose gameplay mode depending on their abilities instead of being randomly selected for each condition, other factors apart from the seated or standing position could have affected the results. In addition, the psychological effects experienced could have influenced exertion levels because the seated players rated the exergame as more usable than the standing players. By contrast, another study comparing seated with standing positions when playing an adapted game mat exergame showed that participants had lower energy expenditure, perceived exertion, and heart rate when they played seated than when they played standing [65]. This aligns with research outside of the exergame domain that shows that performing an otherwise sedentary activity while standing requires more energy expenditure than performing the same activity while seated [66,67]. However, when we compared upper body movement interactions in seated and standing positions for a casual exergame, we found that there were no significant differences in perceived exertion between seated and standing gameplay [41]. Furthermore, an additional study compared playing 8 to 10 minutes of a boxing exergame in standing and seated positions and found that the seated position resulted in lower energy expenditure than the standing position, while there was no significant difference in perceived exertion [68]. As “Every move counts towards better health” according to the World Health Organization [69], even if exergame activities do not exceed recommended intensity levels, playing exergames is superior to being inactive [39] and might lead to higher exercise adherence [70]. Furthermore, microbreaks with light-intensity exercise every 30 minutes during the day offer both physical and mental health benefits [20]. Therefore, it could be argued that playing upper body casual exergames while seated is better than not playing at all.

In terms of psychological effects, research shows that both standing and seated gameplay generally have the same level of enjoyment [41,64,65,71,72]. However, in 3 of these previous studies [64,65,72], no statistical analysis comparing sitting and standing positions was performed, and in 2 of the studies, different movements were used in the sitting and standing conditions [65,71]. Thus, the generalizability of the results regarding the effect of standing versus sitting positions on enjoyment is limited. On the basis of the limitations of previous studies, we statistically compared 2-minute seated and standing casual exergame play with the same movement interactions in a previous study and found no significant difference between playing seated or standing in terms of positive affect and enjoyment [41].

### Objectives

This work is an extension of a conference paper on full body and upper body movement interactions [45]. The aim of this work was to study the psychological effects and physical exertion of playing short-duration casual exergames lasting 2 minutes based on upper body movement interactions designed to promote active microbreaks to interrupt sedentary behavior. Considering the limited research and the mixed results of the psychological effects and physical exertion of different movement interactions in casual exergames, we specifically wanted to explore the psychological effects and physical exertion of upper body compared to full body movement interactions (study 1) and standing compared to seated positions for upper body movement interactions (study 2). The following research questions (RQs) were addressed:

- RQ1: How do casual exergames based on upper body compared to full body movements differ in terms of their psychological effects and physical exertion?
- RQ2: How do upper body casual exergames played in seated positions compared to standing positions differ in terms of their psychological attractiveness and physical exertion levels?

## Methods

### Overview

To answer the RQs, we conducted 2 studies using distinct in-house–developed casual exergames (Crossing and Beaver) for each study. The casual exergames were developed using the open-source game engine Godot. Each round of play lasts 2 minutes. Both casual exergames can be played on a computer screen equipped with a webcam that captures players’ movements. To enable players to observe their movements in relation to on-screen events, the webcam feed is displayed in the top left corner of the casual exergame interface. The actual time and collected points are displayed in the upper right corner of the interface.

Both studies were performed at a game and cosplay festival. People walking by were asked whether they wanted to participate in a study about exergames. Those interested were provided with detailed information, and if willing to participate, they read through the information materials and signed a consent form. Participants aged <18 years needed their guardian to sign the form on their behalf. By recruiting participants who were naturally interested in games, we aimed to evaluate the impact of exergames on a population that is likely to engage with such interventions in real-world settings. This setting provided a unique opportunity to observe the effects of exergames on individuals already motivated by gaming, which we believe is crucial for understanding the design and immediate effects of these interventions.

### Study 1: Upper Body Versus Full Body Movement Interactions

#### Casual Exergame (Crossing): Design and Interaction

**Figure 1:**
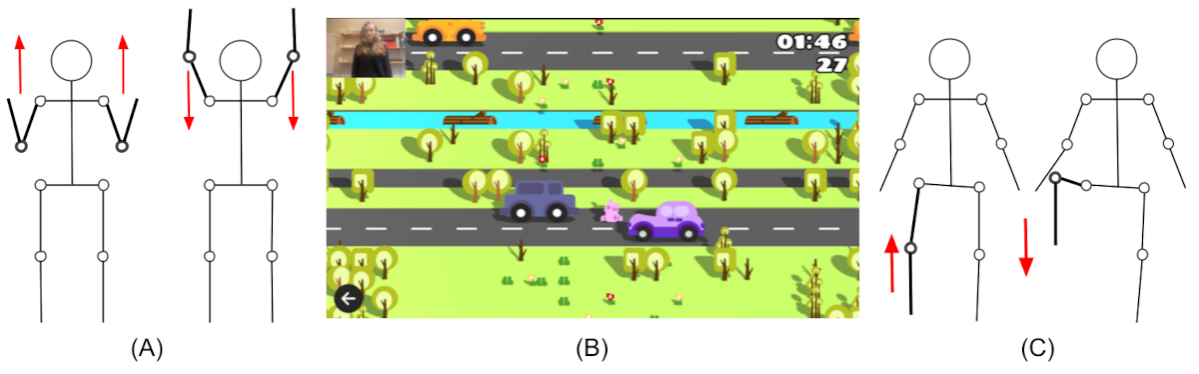
(A) Demonstration of forward movement for upper body movement interactions for (B) the casual exergame Crossing. (C) Demonstration of forward movement for full body movement interactions. Arrows show the direction of movement.

The casual exergame Crossing is based on the classic arcade game Frogger (Figure 1). Using a well-known game genre and basic game mechanics is known to make it easier for players to identify and comprehend the game mechanics [73,74]. In Crossing, players control a rabbit navigating roads, rivers, and rail lines, earning points for forward jumps. Players can also perform sideways jumps, which aid in avoiding obstacles but do not contribute to the accumulation of points. If players collide with vehicles or fall into rivers, the rabbit dies, prompting a 5-second restart. The points acquired before each death are retained, but the waiting period during the restart impacts overall performance.

To support our research, 2 different movement controllers were developed for Crossing: an upper body condition and a full body condition. In the upper body condition, players raise their hands from near their bodies up to their shoulders and above their heads, starting with their arms stretched downward. The rabbit on the screen leaps forward as soon as the hands cross over the head. The player must repeat the movement and extend their arms to the starting position to make the rabbit jump again. In the full body condition, players raise their left or right knee over the hip to make the rabbit jump forward. The rabbit jumps forward when the knee is lifted above the hip. The player must stretch their leg back down to the starting position and repeat the maneuver with either leg to make the rabbit jump once more. To make the rabbit jump to the side in both upper body and full body conditions, players extend a single arm corresponding to the intended direction—raising the right arm for a jump to the right and the left for a jump to the left, unlike extending both arms upward.

Previous research has indicated the importance of shifting the player's focus away from the physical exertion they are experiencing while playing the exergame [58,73]. When it comes to body focus, Crossing’s fast-paced, time-limited gameplay forces players to pay attention to their movements and prevents them becoming self-conscious. The game’s obstacles might *kill* players if they remain motionless for an extended period of time, further contributing to the game’s fast pace [73].

#### Participants and Procedure

In all, 80 people participated in the study. The participants’ ages ranged from 11 to 54 (mean 25, SD 7) years. Of the 80 participants, 56 (70%) identified as man, 17 (21%) as woman, and 5 (6%) as nonbinary, while 2 (3%) reported that they were unsure about their gender identity. Of the 80 participants, 51 (64%) reported having played exergames multiple times, 25 (31%) reported having played exergames once, and 4 (5%) reported never having played exergames.

**Figure 2:**
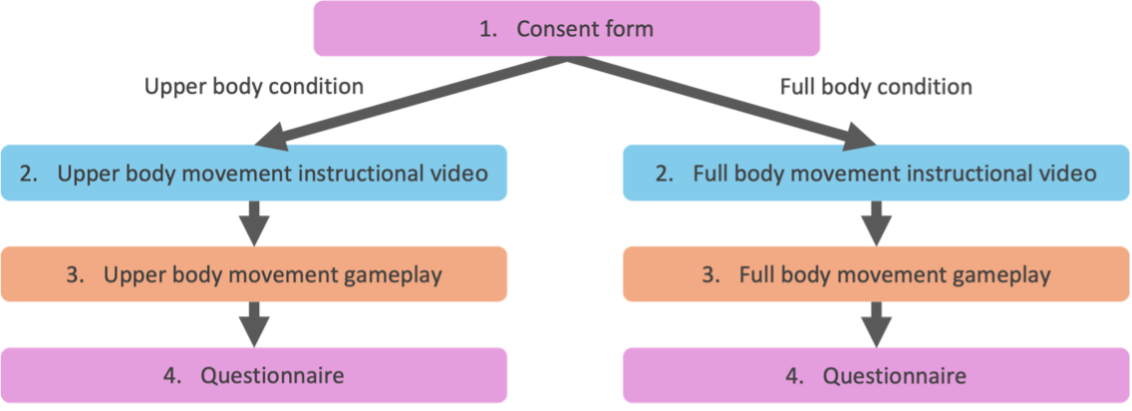
Study 1 procedure.

After signing the consent form, all participants watched an instructional video about how to play and interact with the exergame. Participants were then asked to stand 1.5 meters away from a computer screen equipped with a webcam. The webcam was adjusted so that the participant was clearly visible, and they were asked to demonstrate the movements they should use. Next, participants played one 2-minute round and then filled out a questionnaire (Figure 2) that included (1) demographic questions, (2) the Borg rating of perceived exertion (RPE) scale [40], and (3) the perceived immersion and control subscales of the Exergame Enjoyment Questionnaire (EEQ) [44]. We alternated between controllers for each new participant, resulting in half the participants (40/80, 50%) playing Crossing in the upper body condition and the other half (40/80, 50%) playing in the full body condition.

#### Measures

Physical exertion and psychological effects were the main measures in the study. To measure the physical exertion of playing Crossing, the Borg RPE scale was used to measure perceived exertion [75,76] because it is a valid instrument for measuring exercise intensity [76] and has been used previously to measure perceived exertion when playing casual exergames [33]. The Borg RPE scale ranges from 6 (indicating no exertion at all) to 20 (indicating maximal exertion) [77], with ratings of 11 to 12 corresponding to light intensity, 13 to 14 corresponding to moderate intensity, and 15 to 16 corresponding to hard intensity [78] (Table 1).

**Table 1:** Correspondence between the Borg rating of perceived exertion (RPE) score and percentage of maximum heart rate [78-80].

Maximum heart rate (%)	Intensity	Borg RPE score
20-39	No exertion at all	6-7
40-59	Very light	8-10
60-69	Light	11-12
70-79	Moderate (somewhat hard)	13-14
80-89	Hard (heavy)	15-16
90-99	Very hard	17-18
100	Maximal	19-20

To measure psychological effects, the perceived immersion and control subscales of the EEQ were used (Table 2) [81]. The EEQ perceived immersion subscale consists of 5 items and measures the degree to which the player is fully engaged and involved in the activity [81]. The EEQ perceived control subscale consists of 4 items and measures the degree to which players can directly affect the outcome of the exergame [81]. All items were measured on a 5-point Likert scale: strongly disagree (score=1), disagree (score=2), neutral (score=3), agree (score=4), and strongly agree (score=5).

**Table 2:** The Exergame Enjoyment Questionnaire (EEQ) perceived immersion and control items [81].

Item	Scale	Item wording
Immersion 1^a^	EEQ immersion	I did not feel like I wanted to keep playing.
Immersion 2	EEQ immersion	I felt like I lost track of time while playing.
Immersion 3	EEQ immersion	I felt a strong sense of being in the world of the game to the point that I was unaware of my surroundings.
Immersion 4	EEQ immersion	I felt emotionally attached to the game.
Immersion 5	EEQ immersion	I was focused on the game.
Control 1	EEQ control	I felt that it was easy to familiarize myself with the game controls.
Control 2^a^	EEQ control	I felt that it was difficult to understand how the game works.
Control 3	EEQ control	I felt in control over the game.
Control 4	EEQ control	I felt that the game reacted quickly to my movements.

^a^Reversed item.

To provide descriptive data, the number of movements during the play session was measured by counting the number of movements each participant executed while playing the exergame, encompassing both the forward and sideways jumping movements for the rabbit. In addition, each participant’s performance was measured by recording their final score.

#### Statistical Analysis

A between-subjects analysis was conducted for the players in the upper body condition compared to those in the full body condition using SPSS software (version 29.0; IBM Corp). Before the analysis, the items immersion 1 and control 2 were reversed, and internal consistency was measured for the perceived immersion and control subscales of the EEQ using Cronbach α. The Cronbach α value for the 5-item perceived immersion subscale was 0.66, and the Cronbach α value for the 4-item perceived control subscale was 0.68, both close to the satisfactory threshold value of 0.70 [82]. As several of the variables did not meet the assumption of normality and had no significant outliers [83], we chose to use the nonparametric Mann-Whitney *U* test to answer RQ1. The condition was used as the independent variable and perceived control, perceived immersion, and perceived exertion as the dependent variables. Approximate values of the effect size *r* were calculated by dividing the z score for each test by the square root of the number of cases (n=80) [84], following the guidelines of 0.2 corresponding to a small effect size, 0.5 to a medium effect size, and 0.8 to a large effect size [85]. To complement the analysis, a Spearman correlation matrix was produced looking at the correlation between perceived control, perceived immersion, and perceived exertion for both conditions, following the guidelines of ≤0.35 corresponding to a weak correlation, 0.36 to 0.67 corresponding to a moderate correlation, and 0.68 to 1 corresponding to a strong correlation [86].

### Study 2: Standing Versus Seated Positions for Upper Body Movement Interactions

The second study replicated our previous study on seated and standing exergames [41], using a different casual exergame and with the addition of using the participant’s actual heart rate as an objective measure of exertion.

#### Casual Exergame (Beaver): Design and Interaction

**Figure 3:**
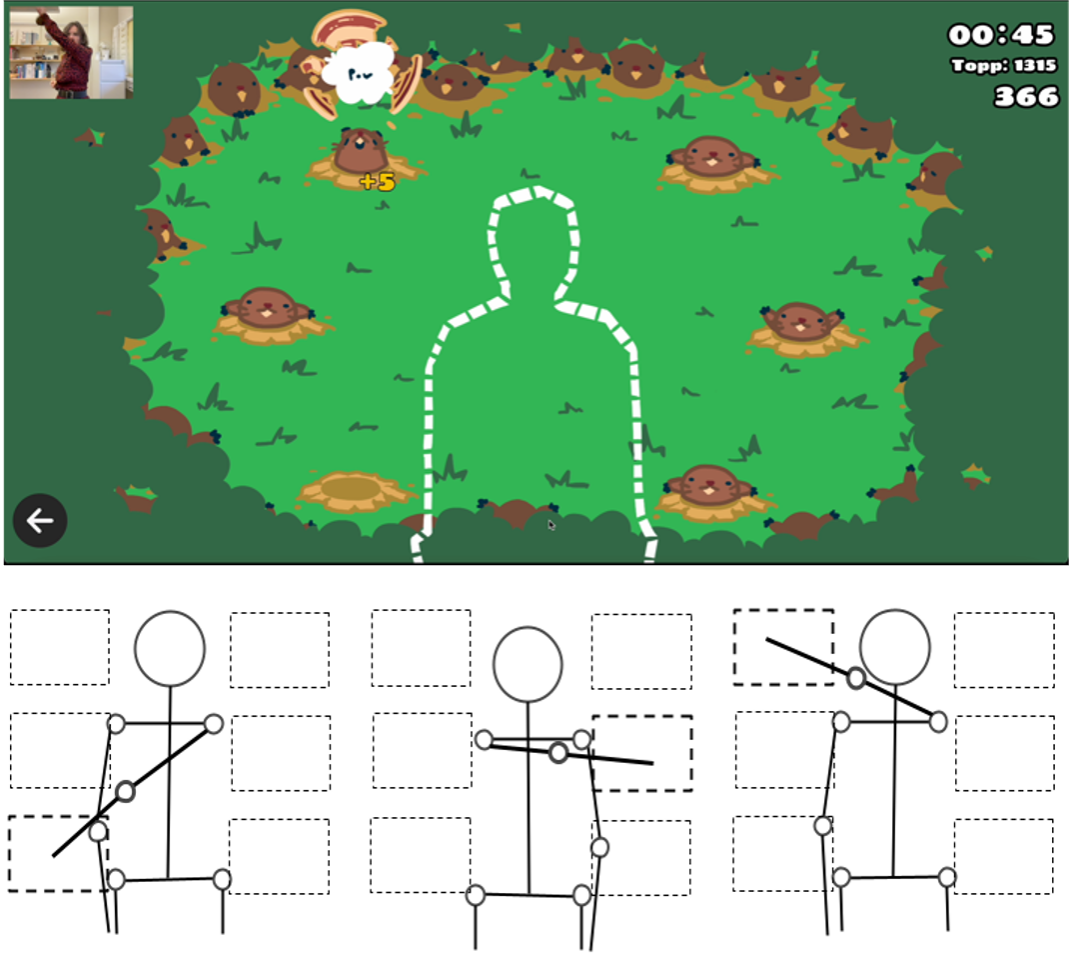
The casual exergame Beaver with boxing-like upper body movement interactions.

The in-house–developed casual exergame Beaver was used in this study. The goal of Beaver is to hit targets on the screen as fast as possible within the time limit of 2 minutes. In the exergame, beavers holding target boards appear on the screen in 6 different positions according to a specific pattern. The player receives points based on how fast the target is hit (slow hit=1 point, moderately fast hit=3 points, and very fast hit=5 points). The order in which the targets appear follows different patterns but is repeated over 2 rounds, allowing players to learn the current pattern and hit the targets faster in the second round. To hit the targets, the player moves the arm on the opposite side across the body toward the target’s position, as shown in Figure 3. Targets alternate between the left and right side of the player’s head, shoulders, and hips so that the player keeps hitting the targets across the torso with both arms. This movement is similar to boxing because prior research on boxing exergames has demonstrated that boxing-like movements can result in moderate levels of exertion [39,60-63]. The decision to avoid a boxing game graphics theme was to maintain consistency with the game-like, rather than sport-like, theme and appearance used in the Crossing exergame in study 1.

#### Participants and Procedure

In all, 40 people participated in the study, and their ages ranged from 18 to 47 (mean 25.27, SD 6.357) years. Of the 40 participants, 23 (58%) identified as man, 12 (30%) as woman, and 4 (10%) as nonbinary, while 1 person (2%) reported that they were unsure about their gender identity. Of the 40 participants, 19 (48%) reported as work was their main occupation, 17 (42%) were students, 2 (5%) worked as well as studied, and 2 (5%) had some other main occupation. Of the 40 participants, 4 (10%) had never played an exergame, 17 (42%) had played an exergame at some point, 14 (35%) sometimes played exergames, 4 (10%) played exergames somewhat often, and 1 (2%) often played exergames.

**Figure 4:**
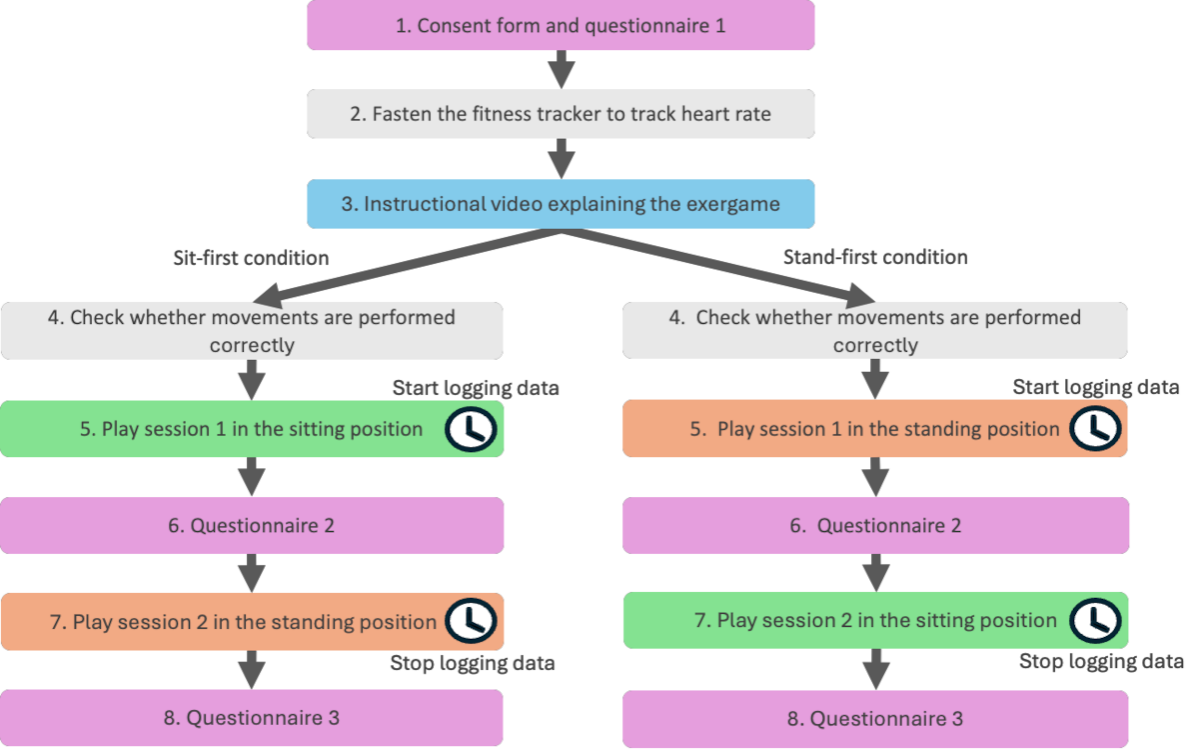
Study 2 procedure.

After signing the consent form, participants answered a questionnaire that included (1) demographic questions and (2) the international, shortened version of the Positive and Negative Affect Schedule (PANAS) [87,88]. A Polar Unite fitness tracker was fastened around each participant’s wrist to track their heart rate during the play sessions. The participants were then shown an instructional video that explained how to play the exergame. To avoid possible bias, the video was recorded in such a way that it was impossible to tell whether the instructor was sitting or standing. Conditions were alternated for each participant, resulting in half of the participants (20/40, 50%) playing Beaver in the sit-first condition (seated in a chair) and the other half (20/40, 50%) playing in the stand-first condition. Participants were placed 1.5 meters away from a computer screen equipped with a webcam in both conditions. The webcam was then adjusted so that the participant was clearly visible, and the participant was asked to demonstrate the movements they should use. Once the participant had confirmed that they had understood the exergame and movements by demonstrating them correctly, the fitness tracker was set to start logging the heart rate, and the participant started the first game session. The time was also noted to correlate the heart rate data to the questionnaire results. After the session ended, the final score was noted, while the participant filled in the second questionnaire, which included (1) the PANAS [87], (2) the Borg RPE scale [40], and (3) the Physical Activity Enjoyment Scale (PACES) [70,89]. Next, the participant was again guided to sit or stand in front of the computer in the position they had not played before. The webcam was adjusted to the new position, and the participant played a second round of the exergame. The starting time of the second gameplay session was again noted for purposes of later identification in the data. When the gameplay session ended, the heart rate data logging was stopped, and the final score of the second gameplay session was noted while the participant filled out the third and final questionnaire, which included the same scales as the second questionnaire. The entire procedure is shown in Figure 4.

#### Measures

Physical exertion of playing exergames can be measured using subjective perception (eg, using the Borg RPE scale [75-77] as in study 1) and objective measures (eg, capturing the actual heart rate) [79]. As there is a strong correlation between heart rate and energy expenditure, heart rate monitors can be used to measure actual exertion in exergames [90]. Borg RPE scores and heart rate measures are positively correlated during exergame play [91]. Thus, integrating both subjective and objective data offers a comprehensive assessment of physical exertion, enhancing accuracy in evaluating exergame physical exertion.

The participant’s actual heart rate was measured using a Polar Unite fitness tracker fastened around their wrist during the gameplay sessions, while the perceived exertion was measured using the Borg RPE scale [75-77], which corresponds to the maximum heart rate [75,78] (Table 1). The fitness tracker logs the wearer’s heart rate during a recorded training session and displays the heart rate over time in a graph. The highest and average heart rates during the first gameplay session and the second gameplay session were compared to the participant’s estimated maximum heart rate (calculated using the following formula: maximum heart rate = 220 – age in years) to obtain a percentage used to determine exercise intensity.

After study 1, some potential issues with measuring immersion in very short exergames were discovered, as described in the Discussion section. Therefore, in study 2, psychological effects were instead measured in terms of affect, enjoyment, and performance. Positive and negative affect before and after playing Beaver were measured using the international, shortened version of the PANAS [88]. The PANAS was selected because of its established reliability and validity in assessing affect [92], its proven efficacy in measuring changes in affect before and after shorter interventions [93], and its frequent use in research on exergames [94]. The shortened PANAS contains 10 validated items—5 for positive affect and 5 for negative affect—and has a total score for both positive and negative affect that ranges from 5 to 25 based on the summation of its items [87]. In this study, only the positive affect scale of the PANAS was analyzed because the negative affect scale exhibited low variation and, as a result, issues with internal consistency, with values close to 0. As the positive and negative affect scales are uncorrelated and independent [88], eliminating the negative affect scale does not impact the findings related to the positive affect scale.

Physical activity enjoyment was measured using the shortened version of the PACES [70,89]. Five of the original 18 statements are included in the shortened version, which has demonstrated strong reliability for exergames across age groups [70]. In the PACES, the player rates how much they agree with each statement using a 7-point Likert scale.

Finally, performance was measured using the participants’ final scores. These scores indicated the number of times the participant had successfully jumped forward on the path.

#### Statistical Analyses

Two main statistical analyses were conducted. First, a within-subjects comparison of the difference in positive affect before and after playing the first round was carried out for both the participants playing the first gameplay session seated and the participants playing the first gameplay session standing. The second analysis was a within-subjects comparison of the differences in heart rate, perceived exertion, affect, enjoyment, and performance between the seated and standing positions for each participant.

For the first analysis, Cronbach α was calculated for positive and negative affect before and after the first gameplay session for participants playing the first gameplay session standing and seated. Internal consistency was acceptable for positive affect before playing for both seated (0.67) and standing (0.75) gameplay, but while negative affect had acceptable consistency for seated (0.70) gameplay, it was low for standing gameplay (0.22). After playing 1 session of the exergame, the internal consistency for positive affect was high for both seated (0.83) and standing (0.86) gameplay, but, once again, the consistency for negative affect was low for standing (0.15) gameplay while remaining high for seated (0.89) gameplay. Because of the low internal consistency for negative affect, only positive affect was analyzed. To evaluate the difference in positive affect after playing 1 session of the exergame, the difference between the positive affect before and after playing the exergame was calculated. The assumption behind the dependent 2-tailed *t* test was checked in terms of outliers and normality. No outliers were found for either sitting or standing gameplay, and the Shapiro-Wilk test statistic showed that the standing group had no significant deviation from normality at a significance of *P*=.94. However, the seated group deviated from normality with a significance of *P*=.04, which is less than the threshold of *P*=.50. Therefore, the nonparametric Wilcoxon signed rank test was run on the positive affect before and after the first seated and standing gameplay sessions. Effect size was calculated using the formula *r* = *z*/sqrt(N) [84].

For the second analysis, the differences between each participant’s seated and standing gameplay were calculated. Cronbach α was calculated for the PACES (seated=0.83 and standing=0.83) as well as positive (seated=0.89 and standing=0.88) and negative (seated=0.78 and standing=0.73) affect. As all scales showed high internal consistency, their items were summed. Next, the differences between standing and seated values were calculated for the PACES, positive and negative affect, Borg RPE, percentage of maximum heart rate reached (highest and average), and game score for each participant and analyzed to check assumptions of normality. Negative affect had multiple extreme outliers and was heavily skewed with a Shapiro-Wilk significance of <.001 and was thus excluded from further analysis. No other score showed deviation from normality according to the Shapiro-Wilk test statistic (positive affect=.13, PACES=.50, Borg RPE=.07, highest heart rate percentage=.69, average heart rate percentage=.39, and game score=.50). While the game score had 1 outlier, it was not extreme, and we decided to keep it for analysis due to its being a possible increase in score between gameplay sessions. The dependent *t* test could thus be used to analyze the differences between standing and seated gameplay.

### Ethical Considerations

According to the Swedish Ethical Review Act [95], this study did not require ethical review, as it posed no apparent risk of physical or psychological harm to the research subjects, did not involve a physical intervention, and did not involve sensitive personal data. Nonetheless, all procedures adhered to ethical standards outlined in Swedish law (SFS 2003:460). Participants were verbally invited to join the study and were provided with an overview of the project and study procedures. Upon agreeing to participate, individuals received a written information letter and were asked to sign the written consent form. Participants were informed of their right to withdraw from the study at any time without providing a reason. To ensure anonymity, each participant was assigned a unique code that could not be traced back to them. Only the project team had access to these coded data.

## Results

### Study 1: Upper Body Versus Full Body Movement Interactions

Overall, the participants perceived the exergame as controllable and immersive, with perceived exertion corresponding to a low-intensity activity for both the upper body and full body movement interactions (Table 3). In the upper body condition, participants on average agreed that the casual exergame was controllable (median 4.00, IQR 0.88) and were between being neutral and somewhat agreeing that the casual exergame was immersive (median 3.40, IQR 0.90), with a perceived exertion corresponding to a low-intensity activity (median 11.00, IQR 2.00). In the full body condition, participants perceived the exergame as less controllable (median 3.50, IQR 1.00) than, and as immersive (median 3.40, IQR 1.00) as, the upper body condition, with a perceived exertion corresponding to a moderate-intensity activity (median 13.00, IQR 3.50). The statistical analysis (Table 4) showed that the exergame was perceived as significantly more controllable in the upper body condition than in the full body condition (*U*=589.00; *P*=.04), corresponding to a small effect size (0.23). However, there was no statistically significant difference between the conditions regarding perceived immersion (*U*=754.00; *P*=.66) and perceived exertion (*U*=652.00; *P*=.15).

**Table 3:** Descriptive statistics for upper body and full body movement interactions.

Measure	Upper body			Full body	
	Mean (SD)	Median (IQR)	Mean (SD)		Median (IQR)
Sideways movements, n	12.88 (6.57)	11.50 (7.50)	9.58 (4.86)		9.00 (8.00)
Forward movements, n	62.30 (13.54)	62.50 (16.00)	56.98 (11.38)		57.50 (16.50)
Performance	58.53 (12.93)	58.50 (16.00)	47.60 (13.98)		46.50 (22.50)
Perceived control	3.78 (0.61)	4.00 (0.88)	3.48 (0.69)		3.50 (1.00)
Perceived immersion	3.24 (0.62)	3.40 (0.90)	3.15 (0.65)		3.40 (1.00)
Perceived exertion	11.47 (2.10)	11.00 (2.00)	12.00 (2.60)		13.00 (3.50)

**Table 4:** Differences between upper body and full body movement interactions.

Variable	∆median (UB^a^–FB^b^)	Mann-Whitney *U* test	z score	*P* value	Effect size (*r*)
Perceived control^c^	0.5	589.00	–2.05	.04^c^	0.23
Perceived immersion	0.0	754.00	–0.45	.66	0.05
Perceived exertion	−2.0	652.00	–1.45	.15	0.16

^a^UB: upper body game controller.

^b^FB: full body game controller.

^c^Significant effect at *P*<.05.

The Spearman correlation matrix (Table 5) showed a significant moderate correlation between perceived immersion and perceived control (0.44; *P*<.001). No significant correlation was found between perceived exertion and perceived control (0.07; *P*=.51) and between perceived exertion and perceived immersion (0.11; *P*=.33).

**Table 5:** Spearman correlation matrix of perceived control, immersion, and exertion.

Variable		Perceived control	Perceived immersion	Perceived exertion
**Perceived control**				
	*r*	—^a^	0.44	0.07
	*P* value	—	<.001^b^	.51
**Perceived immersion**				
	*r*	0.44	—	0.11
	*P* value	<.001^b^	—	.33
**Perceived exertion**				
	*r*	0.07	0.11	—
	*P* value	.51	.33	—

^a^Not applicable.

^a^*P* value met the threshold for significance.

### Study 2: Standing Versus Seated Positions for Upper Body Movement Interactions

There was an increase in positive affect scores from before (mean 13.90, SD 3.43; median 14.00, IQR 4.50) to after (mean 16.00, SD 4.61; median 15.00, IQR 7.00) playing one 2-minute session of the exergame in the seated position. The Wilcoxon signed rank test showed that this difference was significant (*z*=–3.288; *P*=.001), and the effect size (*r*=0.74) corresponded to a large effect [85]. There was also an increase in positive affect scores from before (mean 13.45, SD 3.69; median 13.50, IQR 5.00) to after (mean 16.00, SD 4.19; median 16.00, IQR 7.00) playing one 2-minute session of the exergame in the standing position. The Wilcoxon signed rank test showed that this was also significant (*z*=–2.947; *P*=.003), and the effect size (*r*=0.66) corresponded to a large effect [85].

The highest percentage of their maximum heart rate that participants reached during standing gameplay (mean 63.29%, SD 7.40%) was slightly higher than that reached during seated gameplay (mean 60.46%, SD 7.38%), which was significant (t_39_=2.805; *P*=.008), with an effect size (Cohen *d*=0.44) corresponding to a small effect. Similarly, the average heart rate (as a percentage of the estimated maximum heart rate) was also slightly higher in standing gameplay (mean 55.71%, SD 7.78%) than in seated gameplay (mean 52.63%, SD 6.92%). This was again statistically significant (t_39_=2.542; *P*=.02), with an effect size (Cohen *d*=0.40) corresponding to a small effect.

Standing gameplay also showed slightly higher scores in positive affect, enjoyment, and perceived exertion than seated gameplay, while seated gameplay had slightly higher scores in performance than standing gameplay. However, none of these differences were significant (Table 6).

**Table 6:** Statistical analysis of seated gameplay compared to standing gameplay.

Measure	Seated gameplay, mean (SD)	Standing gameplay, mean (SD)	Seated gameplay, median (IQR)	Standing gameplay, median (IQR)	*t* test (*df*)	*P* value	Effect size (Cohen *d*)
Positive affect	15.80 (4.85)	16.13 (4.82)	15.00 (8.50)	15.50 (7.00)	1.114 (39)	.27	0.18
Enjoyment	25.38 (5.67)	25.90 (5.66)	25.00 (9.50)	25.50 (8.50)	0.894 (39)	.38	0.14
Perceived exertion	10.60 (2.04)	10.95 (2.40)	11.00 (3.00)	11.00 (3.00)	1.300 (39)	.20	0.21
Average heart rate (%)	52.63 (6.92)	55.71 (7.78)	51.93 (1.74)	55.21 (0.97)	2.542 (39)	.02^a^	0.40
Highest heart rate (%)	60.46 (7.38)	63.29 (7.40)	60.21 (8.44)	62.24 (8.93)	2.805 (39)	.008^a^	0.44
Performance	702.30 (109.52)	695.50 (94.13)	716.50 (154.50)	706.00 (116)	–0.409 (39)	.69	0.07

^a^*P* value met the threshold for significance.

## Discussion

This work aimed to study the psychological effects and physical exertion of playing short-duration casual exergames lasting 2 minutes based on upper body movement interactions designed to promote active microbreaks for people who are sedentary. In the first study, upper body movement interactions were compared to full body movement interactions, and in the second study, seated gameplay was compared to standing gameplay when using upper body movement interactions.

### Principal Findings

This work demonstrated that (1) upper body movement interactions in casual exergames can be as effective and appealing as full body movement interactions; and (2) playing upper body casual exergames in standing positions can result in slightly higher effectiveness than, and a similar degree of attractiveness as, playing upper body casual exergames in seated positions.

In terms of the psychological effects of casual exergames, study 1 showed that the upper body casual exergame controller was perceived as more controllable than the full body controller and that the level of immersion was similar for both conditions. As perceived control is seen as a prerequisite to an immersive experience [50] and was found to positively correlate to immersion in this study, longer-duration gameplay could have resulted in the upper body condition being perceived as more immersive over time; for example, 1 study found that short-duration gameplay lasting 3 minutes resulted in less immersion than long-duration gameplay lasting 7 minutes [96]. The short-duration gameplay session lasting 2 minutes in this study could thus have resulted in lower immersion than if the exergame had been played for longer durations. Further research would be needed to investigate whether this holds true. In study 2, the psychological effects were similar for the seated and standing upper body movement conditions, with no significant difference in positive affect, enjoyment, or performance. Furthermore, the results showed that both seated and standing upper body casual exergame movement interactions significantly increased positive affect after participants played a 2-minute session. The result signifies that seated and standing casual exergame play could both be valid movement interaction options for upper body casual exergames.

In terms of physiological effectiveness, study 1 showed that perceived exertion did not differ for the upper body and full body casual exergame conditions. Similarly, in study 2, there was no significant difference between perceived exertion for the standing and seated upper body casual exergame conditions. However, there was a difference in terms of the objective measure of exertion. The standing group had a significantly higher heart rate than the sitting group for both the highest and average heart rates, although the effect size was small for both. As no objective measure of exertion was taken in study 1, a similar pattern could have emerged for the upper body conditions compared to the full body conditions. The upper body movement interactions provided a light level of perceived exertion in both study 1 (mean 11.47, SD 2.10), with the upper body movement corresponding to players lifting their arms, and study 2 (mean 10.60, SD 2.04 for seated gameplay and mean 10.95, SD 2.40 for standing gameplay), with the upper body movement corresponding to boxing-like movements. However, while the highest heart rates reached also showed a light level of exertion (mean 60.46%, SD 7.38% for seated gameplay and mean 63.29%, SD 7.40% for standing gameplay), the average heart rates in study 2 (mean 52.63%, SD 6.92% for seated gameplay and mean 55.71%, SD 7.78% for standing gameplay) only indicate a very light–intensity activity, which might also be the case for study 1. On the basis of previous research claiming that casual exergames should produce at least a moderate level of exertion [33], neither exergame thus reached sufficient levels of exertion. Despite this, both exergames may be considered appropriate tools for microbreaks during the day, considering that every move contributes to better health [69], and microbreaks (2-3 min of light-intensity exercise) during the day (every 30 min) still offer both physical and mental health benefits [20-22]. The higher heart rate averages displayed in study 2 imply that people could gain some benefit from playing upper body casual exergames in standing positions compared to seated positions. However, due to the small difference in effect size and with both measures corresponding to very light activity, playing seated should be seen as a reasonable option for those who prefer to do so. Further research is necessary to ascertain the long-term effects on physical health of playing in seated or standing positions.

### Comparison With Prior Work

The importance of considering both the psychological effects and the physical exertion of playing exergames has been highlighted previously [46-48, 73]. This study focused on how the movement interactions used in casual exergames could affect these dimensions.

Previous research has shown that playing exergames in both standing and seated positions produces equivalent levels of enjoyment (psychological attractiveness) [41,64,65,71,72]. The results from this work further support these findings, showing no difference between the seated and standing positions in terms of enjoyment; in addition, they support the notion that the psychological effects are also similar in terms of positive affect and performance [41]. Previous research has also shown that playing casual exergames can induce positive affective states [31,32,41]. The results of this study support this notion, with both the seated and standing positions generating an increase in positive affect. As positive affect can reduce stress levels [42], enhance overall well-being [43], and improve work performance [97], casual exergames could be promising for implementation in a workplace context to promote active microbreaks.

Regarding physiological effectiveness, previous studies have found mixed results when comparing standing and seated exergaming. While some studies have found higher energy expenditure in seated gameplay [64,65], others have found higher energy expenditure in standing gameplay [68,72]. Our results in study 2 support that standing gameplay involves higher objective exertion than seated gameplay, with no difference in subjective measures. The higher exertion in the standing position is likely due to using the leg muscles to stand (although they are not used to play the exergame) because even sedentary activities require more energy when standing instead of sitting [66,67]. When rating perceived exertion, this difference might be small enough that people do not register it because the exertion in their arms is more noticeable after playing, thus leading to similarly rated exertion despite differing heart rates. The results show that the difference in heart rate percentage is only approximately 3% for both the highest and average heart rates, which may feel very similar. As for why the participants rated their exertion higher than their heart rates show, it is possible that the average over the 2 minutes of gameplay does not reflect the exertion level at the end, which is when the participants rated their exertion. When beginning an activity, the heart rate rises gradually. With the short playtimes, this initial increase might affect the average heart rate more than it would in a longer activity. The highest heart rate reached was typically toward the end of the sessions and was also more closely matched to the perceived exertion.

Previous studies have also shown mixed results when comparing upper body to lower body movement interactions. It has been suggested by some studies that upper body exergaming might not be sufficient for achieving adequate energy expenditure [46,59]. Meanwhile, other studies have suggested that certain upper body movements could produce sufficient exertion levels [39,60-63]. This study found no difference in perceived exertion between upper body and full body movement interactions, somewhat supporting the notion that upper body movements could produce sufficient exertion. However, because no objective measures of exertion were recorded in study 1, more research would be needed to validate that this holds true not only for subjective measures of exertion but also for objective measures.

A meta-review shows that upper body movement interactions characterized by continuous movements can result in greater energy expenditures and intensity levels [39]. Previous studies on boxing exergames have shown that boxing-like movements can achieve a moderate intensity of exertion [39,60-63]. However, both the perceived exertion and the heart rate data showed that the exergame in study 2, which involved using boxing-like movements, only reached a light or very light intensity of exertion. A potential explanation for this difference could be the time participants spent playing because a 2-minute exergame session will feel less exerting than the same exergame played for twice as long or more. Another explanation could be that the design of the exergame influenced the amount of physical effort the participants were willing to exert; for example, the exergame in study 2 features short pauses between sets of targets, which could have contributed to participants not reaching moderate levels of exertion. The interplay between gameplay duration, movement design, and exergame design is an interesting avenue for further research.

### Limitations

Integrating active microbreaks into the day via casual exergames has shown promising advantages [32,37]. However, both studies took place at a game and cosplay festival at a specific point in time. The 2-minute casual exergames may influence positive affect in distinct ways when experienced in other settings; for example, having an audience of peers sharing an interest in gaming might be experienced as more positive than playing alone. Furthermore, because the number of people observing the players varied throughout the day depending on other activities at the festival, participants may also have had different experiences depending on their reactions to feeling observed. Conducting the studies in a more controlled setting might produce different results. In addition, continuous gameplay might play a role in influencing changes in positive affect over time. Further research should also be undertaken to quantify the findings of these studies by using other casual exergames.

Participants in both studies played the exergames for the first time. Thus, the lack of familiarity may have influenced their impression of the movement interactions in both study 1 (upper body movement interactions compared to full body movement interactions) and study 2 (upper body movement interactions in seated compared to standing positions). Further research should incorporate a phase allowing participants to familiarize themselves with the exergame controllers before the start of the study. This would help determine whether the findings remain applicable when participants have fully mastered the exergame controls. As this study only covered players’ initial experience with casual exergames, there is a need for longitudinal studies on the psychological attractiveness and physiological effectiveness of different movement interactions in casual exergames and how they should be constructed to increase the psychological attractiveness and physical effectiveness over time.

The results regarding exertion in study 1 are also limited due to a lack of objective measures of exertion. As seen in study 2, there could be differences in the full body and upper body movement interactions in terms of exertion that are not captured through only perceived exertion. Further research using objective measures (eg, heart rate) should be undertaken to better understand the effectiveness of full body and upper body movement interactions in short casual exergames. Furthermore, both studies could greatly benefit from using more extensive measures of physical activity, such as metabolic equivalents of tasks (METs) and maximal oxygen consumption, to examine the extent of any health benefits of playing short-duration casual exergames in different player modes.

### Conclusions

Short-duration casual exergames lasting 2 minutes with upper body movements may hold potential in promoting active microbreaks during sedentary periods. As this study showed, upper body casual exergame play can produce light exertion levels and an increase in positive affect after compared to before playing. Upper body movement interactions may also be more suitable for casual exergames used for microbreaks because they are perceived as easier to control than full body movement interactions, while reaching similar (light) exertion levels and immersion. Playing with upper body movement interactions in standing gameplay could involve slightly higher objective exertion (as measured by the heart rate) than when playing the same exergame in a seated position; however, due to the small difference, seated positions should not be neglected as a viable alternative for players who prefer them. Furthermore, upper body movement interactions in both seated and standing positions elicit comparable psychological effects, resulting in similar levels of enjoyment, positive affect, and performance.

## Abbreviations

EEQ: Exergame Enjoyment Questionnaire
MET: metabolic equivalent of task
PACES: Physical Activity Enjoyment Scale
PANAS: Positive and Negative Affect Schedule
RPE: rating of perceived exertion
RQ: research question
